# Supplementary material for: The dynamic role of TRIM8, a novel ciliary protein, during various stages of mitosis
Source: Cell Death Dis. 2025 Oct 7;16(1):707. doi: 10.1038/s41419-025-07973-7 (PMC12504472; doi:10.1038/s41419-025-07973-7)
Supplement: Supplementary file 1 — Supplementary_Figure_Legends [file 41419_2025_7973_MOESM1_ESM.pdf]

**Supplementary Figure 1. Z-score analysis of upregulated proteins from LC–MS/MS following *TRIM8*-silencing.** A positive Z-score indicates increased activation of the associated biological function. *"Cell Cycle Control of Chromosomal Replication"* was identified as the top enriched pathway.

**Supplementary Figure 2. Cellular component analysis of all (both up and down) differentially expressed proteins from the proteomics study.** Analysis using enrichGO.R identified "Ribosome" and "Cytosolic Ribosome" among the top significantly enriched cellular components.

**Supplementary Figure 3. Gene expression patterns of 95 cell cycle marker genes across identified clusters.** Cluster 6 (Phenograph_A1EZ_6) shows higher expression of G2/M phase genes compared to Cluster 2 (Phenograph_A1EZ_2), which itself exhibits greater expression of S-phase and G2/M genes than Cluster 1 (Phenograph_A1EZ_1). Cluster 2 also shows higher expression of S-phase genes than Cluster 6, while Cluster 6 expresses more S-phase and G2/M genes than Cluster 1. In total, 53 G2/M marker genes (e.g., TOP2A) and 42 S-phase marker genes were used for classification. Based on these patterns, Cluster 6 corresponds to the G2/M phase, Cluster 2 to the S-phase, and Cluster 1 to the G0/G1 phase.

**Supplementary Figure 4. Localization of TRIM8 and CEP170 during mitosis in HEK293 and SH-SY5Y cells.** During interphase, TRIM8 displays a scattered nuclear dot-like distribution. Throughout all phases of mitosis, TRIM8 consistently localizes with CEP170, particularly at the centrosomal region.

**Supplementary Figure 5. TRIM8 knockdown impairs ciliogenesis using different siRNAs.** (A) Validation of TRIM8 siRNAs knockdown efficiency by Western blot analysis in RPE cells after 48h of transfection with siRNA Negative control (Ctrl), siRNA TRIM8 HSS129955 (siRNA1), siRNA TRIM8 HSS129956 (siRNA2), siRNA TRIM8 HSS188606 (siRNA3), pool of the three siRNAs TRIM8 (TRIM8i pool). TRIM8 expression level was calculated and graphed normalized to the Ctrl. (B) Primary cilia formation upon TRIM8 silencing. Percentage of ciliated cells was calculated for each condition and graphed (n=2 biological replicates, 300 cells counted per n). (C) Cilia length was measured on IF staining of ARL13B for each condition and graphed (n=2 biological replicates, 50 cilia counted per n). Asterisks indicate statistical significance using unpaired t test, p<0,05, **p<0,0021, *p<0,0002, ***p<0,0001.

**Supplementary Table 1. List of differentially expressed proteins from the LC–MS/MS study and downstream analysis using Ingenuity Pathway Analysis (IPA).**

**Supplementary Table 2. Cluster-specific analysis of differentially expressed genes from scRNA-seq using Ingenuity Pathway Analysis (IPA).**
