# Supplementary material for: The dynamic role of TRIM8, a novel ciliary protein, during various stages of mitosis
Source: Cell Death Dis. 2025 Oct 7;16(1):707. doi: 10.1038/s41419-025-07973-7 (PMC12504472; doi:10.1038/s41419-025-07973-7)
Supplement: Supplementary file 2 — Supplementary Figure 1. Z-score analysis of upregulated proteins from LC–MS/MS following TRIM8-silencing. [file 41419_2025_7973_MOESM2_ESM.pdf]

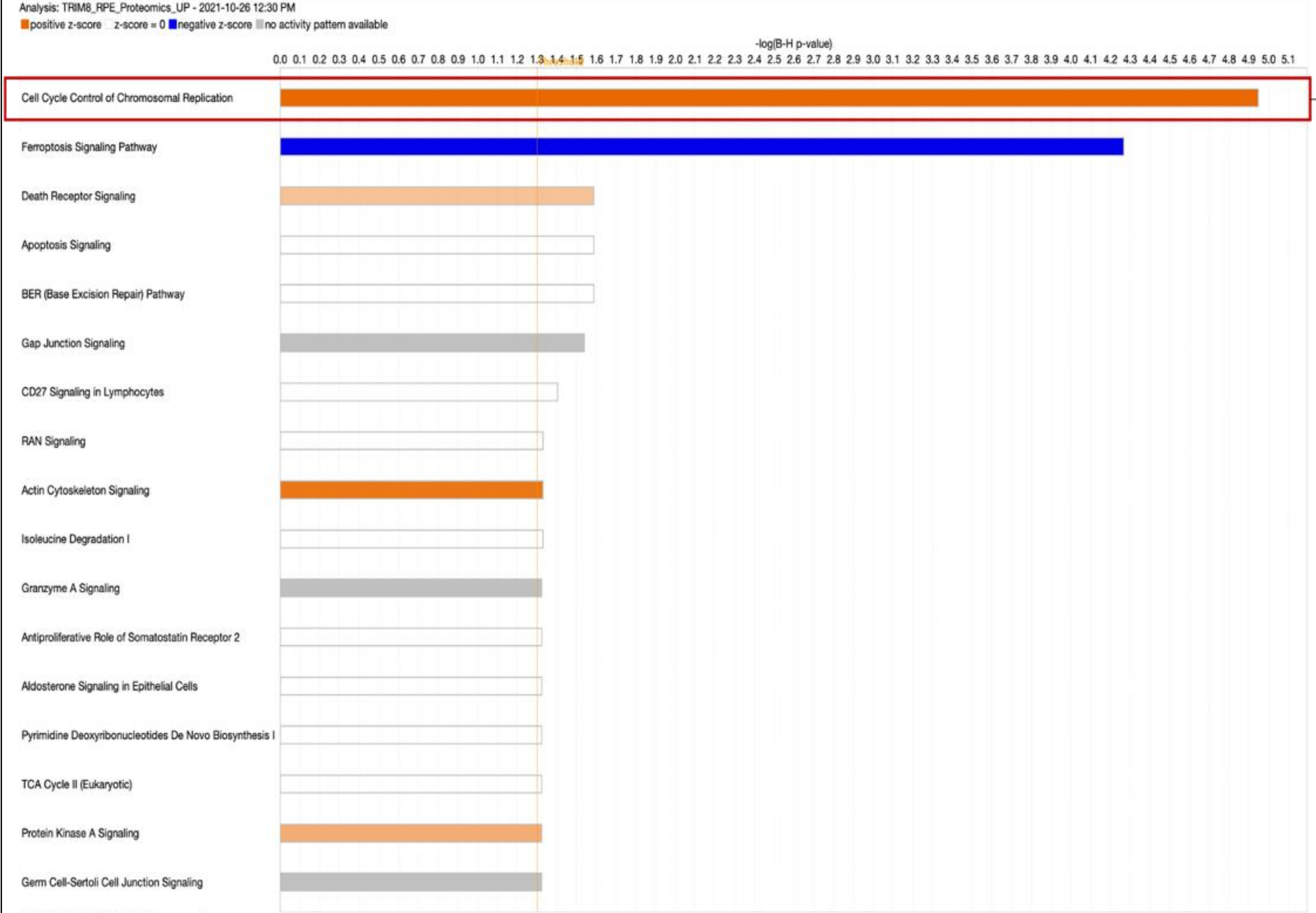

© 2000-2021 QIAGEN. All rights reserved.

"Cell Cycle Control of Chromosomal Replication" is the top hit with a *-log (B-H p-value)* of 4.95 and a positive *z-score* of 2.449 among the canonical pathways.

Note: Positive *z-score* signifes the increase in actvation of biological function.

INGENUITY PATHWAY ANALYSIS
QIAGEN
