# Supplementary figures and images for: The dynamic role of TRIM8, a novel ciliary protein, during various stages of mitosis
Source: Cell Death Dis. 2025 Oct 7;16(1):707. doi: 10.1038/s41419-025-07973-7 (PMC12504472; doi:10.1038/s41419-025-07973-7)

# Cellular components of all DE proteins from the proteomics study

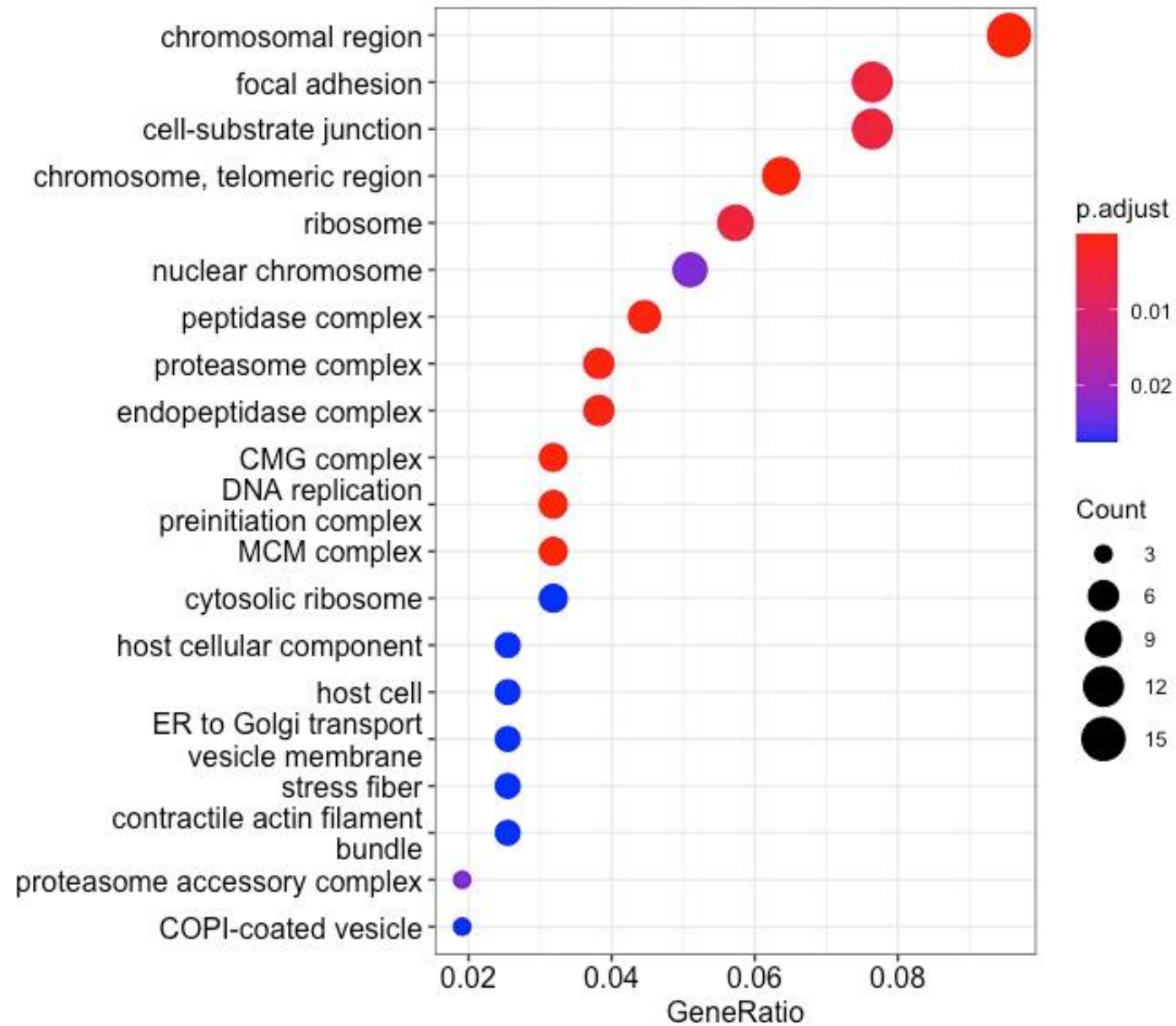

Supplement: Supplementary file 3 — Supplementary Figure 2. Cellular component analysis of all (both up and down) differentially expressed proteins from the proteomics study. [file 41419_2025_7973_MOESM3_ESM.pdf]

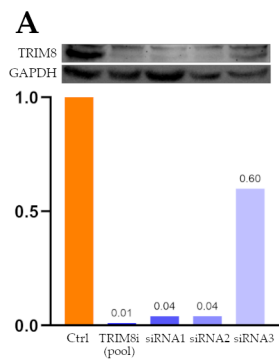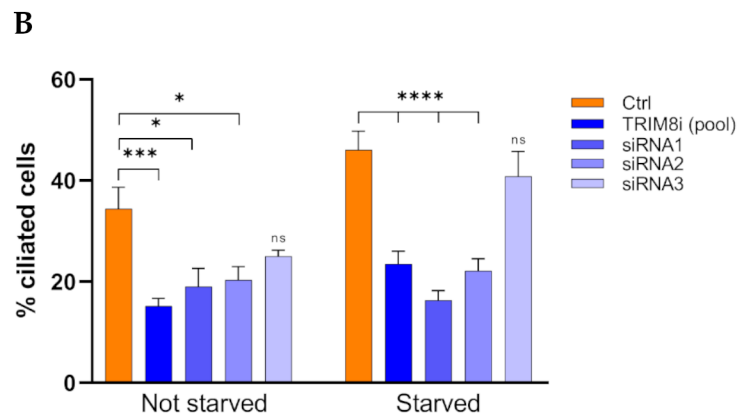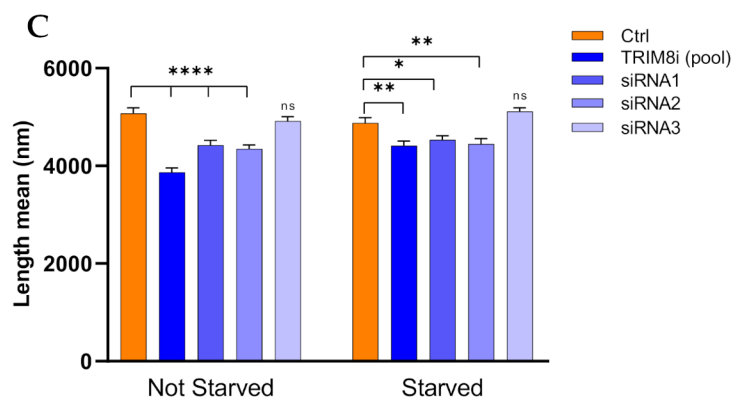

Supplement: Supplementary file 6 — Supplementary Figure 5. TRIM8 knockdown impairs ciliogenesis using different siRNAs. [file 41419_2025_7973_MOESM6_ESM.pdf]
