# Supplementary material for: The dynamic role of TRIM8, a novel ciliary protein, during various stages of mitosis
Source: Cell Death Dis. 2025 Oct 7;16(1):707. doi: 10.1038/s41419-025-07973-7 (PMC12504472; doi:10.1038/s41419-025-07973-7)
Supplement: Supplementary file 4 — Supplementary Figure 3. Gene expression patterns of 95 cell cycle marker genes across identified clusters. [file 41419_2025_7973_MOESM4_ESM.pdf]

Gene expression patterns of 95 cell cycle marker genes among the clusters

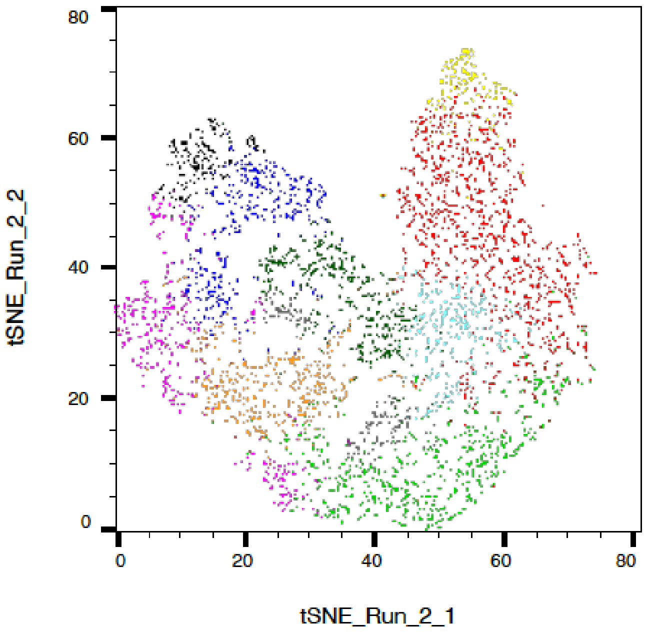

	Subset Name	Count
	Phenograph_A1EZ_1	1238
	Phenograph_A1EZ_2	789
	Phenograph_A1EZ_3	532
	Phenograph_A1EZ_4	519
	Phenograph_A1EZ_5	517
	Phenograph_A1EZ_6	497
	Phenograph_A1EZ_7	377
	Phenograph_A1EZ_8	231
	Phenograph_A1EZ_9	220
	Phenograph_A1EZ_10	189

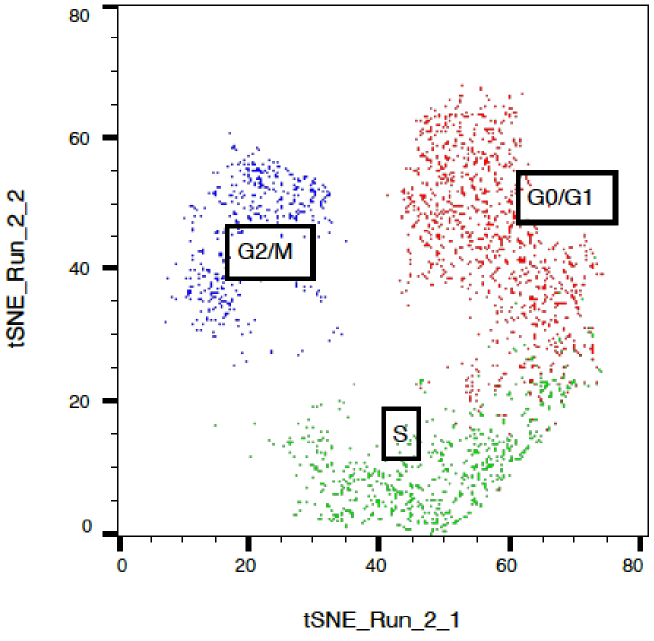

	Subset Name	Count	Mean : TOP2A	Mean : CCNB1	Mean : CCNB2
	Phenograph_A1EZ_1	1238	1.32	0.49	0.42
	Phenograph_A1EZ_2	789	3.75	0.88	0.77
	Phenograph_A1EZ_6	497	27.4	6.08	5.03

	Subset Name	Count	Mean : MCM2	Mean : MCM6	Mean : CCNE2
	Phenograph_A1EZ_1	1238	0.36	0.35	0.16
	Phenograph_A1EZ_2	789	2.19	2.34	1.94
	Phenograph_A1EZ_6	497	1.28	0.90	0.25

Clusters under comparison	UP S-phase genes (42 marker genes)	UP G2/M-phase genes (53 marker genes)	Comments
Cluster 1 vs. Cluster 2	0	0	Cluster 1 has no S- or G2/M-phase genes than cluster 2
Cluster 1 vs. Cluster 6	0	0	Cluster 1 has no S- or G2/M-phase genes than cluster 6
Cluster 2 vs. Cluster 1	37	29	Cluster 2 has more S-phase and G2/M genes than cluster 1
Cluster 2 vs. Cluster 6	17	0	Cluster 2 has more S-phase genes than cluster 6
Cluster 6 vs. Cluster 2	4	50	Cluster 6 has more G2/M phase genes than Cluster 2
Cluster 6 vs. Cluster 1	32	53	Cluster 6 has more S-phase and G2/M phase genes than Cluster 1
