# Supplementary material for: The dynamic role of TRIM8, a novel ciliary protein, during various stages of mitosis
Source: Cell Death Dis. 2025 Oct 7;16(1):707. doi: 10.1038/s41419-025-07973-7 (PMC12504472; doi:10.1038/s41419-025-07973-7)

**A. Localisation of TRIM8 and CEP170 during mitosis in HEK293 cells**

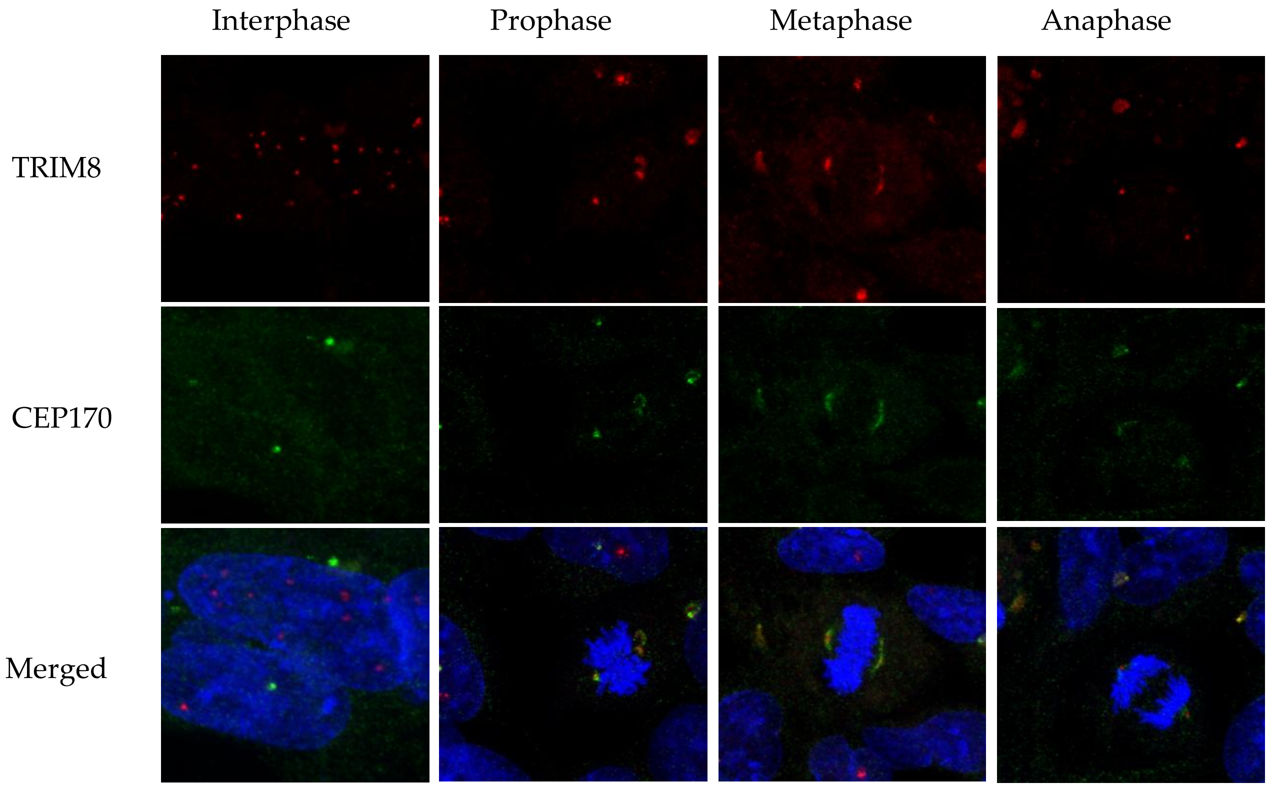


**B. Localisation of TRIM8 and CEP170 during mitosis in SH-SY5Y cells**

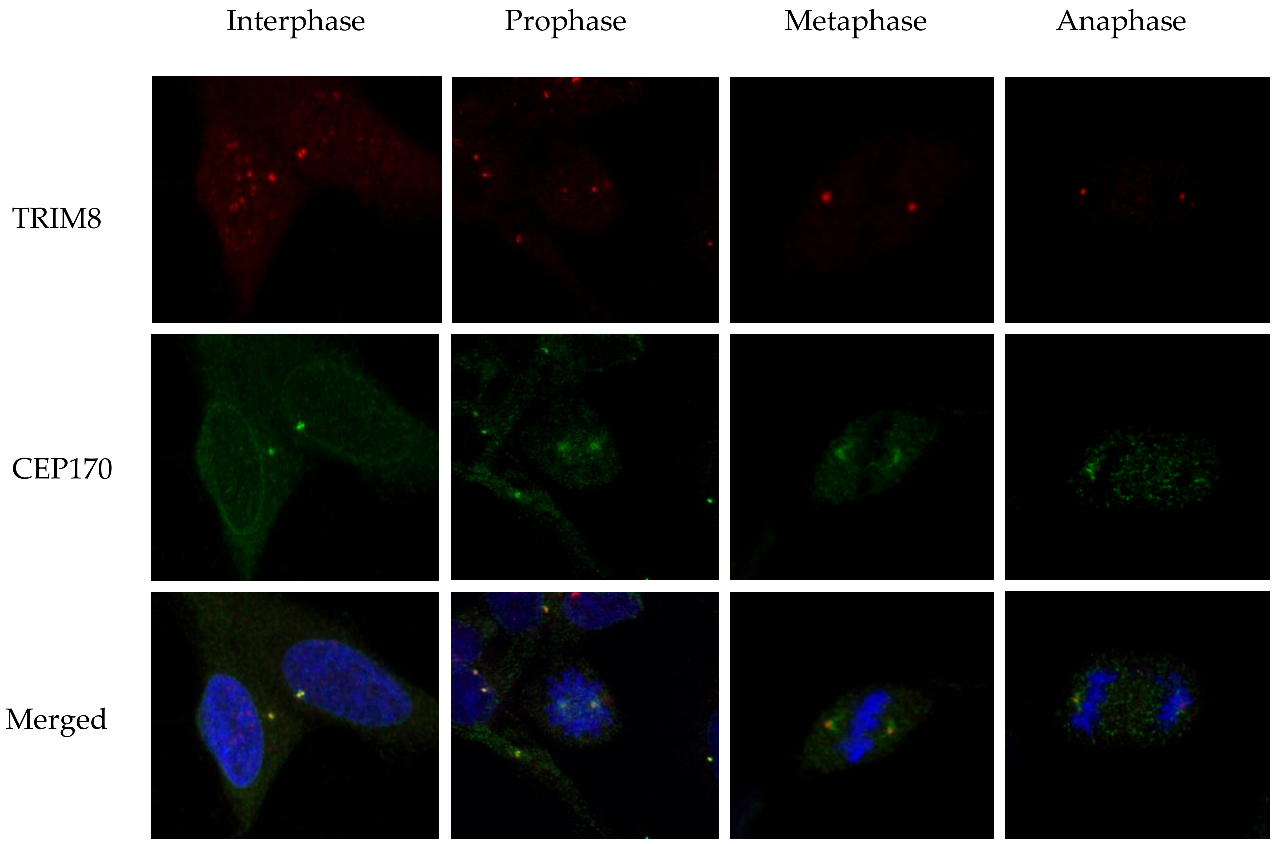

Supplement: Supplementary file 5 — Supplementary Figure 4. Localization of TRIM8 and CEP170 during mitosis in HEK293 and SH-SY5Y cells. [file 41419_2025_7973_MOESM5_ESM.pdf]
